# Guidelines for Developing and Reporting Machine Learning Predictive Models in Biomedical Research: A Multidisciplinary View

**DOI:** 10.2196/jmir.5870

**Published:** 2016-12-16

**Authors:** Wei Luo, Dinh Phung, Truyen Tran, Sunil Gupta, Santu Rana, Chandan Karmakar, Alistair Shilton, John Yearwood, Nevenka Dimitrova, Tu Bao Ho, Svetha Venkatesh, Michael Berk

**Affiliations:** ^1^Centre for Pattern Recognition and Data AnalyticsSchool of Information TechnologyDeakin UniversityGeelongAustralia; ^2^Deakin UniversityGeelongAustralia; ^3^Philips ResearchBriarcliff Manor, NYUnited States; ^4^Japan Advanced Institute of Science and TechnologyNomiJapan

**Keywords:** machine learning, clinical prediction rule, guideline

## Abstract

**Background:**

As more and more researchers are turning to big data for new opportunities of biomedical discoveries, machine learning models, as the backbone of big data analysis, are mentioned more often in biomedical journals. However, owing to the inherent complexity of machine learning methods, they are prone to misuse. Because of the flexibility in specifying machine learning models, the results are often insufficiently reported in research articles, hindering reliable assessment of model validity and consistent interpretation of model outputs.

**Objective:**

To attain a set of guidelines on the use of machine learning predictive models within clinical settings to make sure the models are correctly applied and sufficiently reported so that true discoveries can be distinguished from random coincidence.

**Methods:**

A multidisciplinary panel of machine learning experts, clinicians, and traditional statisticians were interviewed, using an iterative process in accordance with the Delphi method.

**Results:**

The process produced a set of guidelines that consists of (1) a list of reporting items to be included in a research article and (2) a set of practical sequential steps for developing predictive models.

**Conclusions:**

A set of guidelines was generated to enable correct application of machine learning models and consistent reporting of model specifications and results in biomedical research. We believe that such guidelines will accelerate the adoption of big data analysis, particularly with machine learning methods, in the biomedical research community.

## Introduction

Big data is changing every industry. Medicine is no exception. With rapidly growing volume and diversity of data in health care and biomedical research, traditional statistical methods often are inadequate. By looking into other industries where modern machine learning techniques play central roles in dealing with big data, many health and biomedical researchers have started applying machine learning to extract valuable insights from ever-growing biomedical databases, in particular with predictive models [1,2]. The flexibility and prowess of machine learning models also enable us to leverage novel but extremely valuable sources of information, such as wearable device data and electronic health record data [3].

Despite its popularity, it is difficult to find a universally agreed-upon definition for machine learning. Arguably, many machine learning methods can be traced back as far as 30 years ago [4]. However, machine learning started making a broad impact only in the last 10 years. The reviews by Jordan and Mitchell [5] and Ghahramani [6] provide accessible overviews for machine learning. In this paper, we focus on machine learning predictive methods and models. These include random forest, support vector machines, and other methods listed in Multimedia Appendix 1. They all share an important difference from the traditional statistical methods such as logistic regression or analysis of variance—the ability to make accurate predictions on unseen data. To optimize the prediction accuracy, often the methods do not attempt to produce interpretable models. This also allows them to handle a large number of variables common in most big data problems.

Accompanying the flexibility of emerging machine learning techniques, however, is uncertainty and inconsistency in the use of such techniques. Machine learning, owing to its intrinsic mathematical and algorithmic complexity, is often considered a “black magic” that requires a delicate balance of a large number of conflicting factors. This, together with inadequate reporting of data sources and modeling process, makes research results reported in many biomedical papers difficult to interpret. It is not rare to see potentially spurious conclusions drawn from methodologically inadequate studies [7-11], which in turn compromises the credibility of other valid studies and discourages many researchers who could benefit from adopting machine learning techniques.

Most pitfalls of applying machine learning techniques in biomedical research originate from a small number of common issues, including data leakage [12] and overfitting [13-15], which can be avoided by adopting a set of best practice standards. Recognizing the urgent need for such a standard, we created a minimum list of reporting items and a set of guidelines for optimal use of predictive models in biomedical research.

## Methods

### Panel of Experts

In 2015, a multidisciplinary panel was assembled to cover expertise in machine learning, traditional statistics, and biomedical applications of these methods. The candidate list was generated in two stages. The panel grew from a number of active machine learning researchers attending international conferences including the Asian Conference on Machine Learning, the Pacific Asia Conference on Knowledge Discovery and Data Mining, and the International Conference on Pattern Recognition. The responders were then asked to nominate additional researchers who apply machine learning in biomedical research. Effort was exercised to include researchers from different continents. Researchers from the list were approached through emails for joining the panel and/or recommending colleagues to be included. Two declined the invitation.

The final panel included 11 researchers from 3 institutions on 3 different continents. Each panelist had experience and expertise in machine learning projects in biomedical applications and has learned from common pitfalls. The areas of research expertise included machine learning, data mining, computational intelligence, signal processing, information management, bioinformatics, and psychiatry. On average, each panel member had 8.5 years’ experience in either developing or applying machine learning methods. The diversity of the panel was reflected by the members’ affiliation with 3 different institutions across 3 continents.

### Development of Guidelines

Using an iterative process, the panel produced a set of guidelines that consists of (1) a list of reporting items to be included in a research article and (2) a set of practical sequential steps for developing predictive models. The Delphi method was used to generate the list of reporting items.

The panelists were interviewed with multiple iterations of emails. Email 1 asked panelists to list topics to be covered in the guidelines. An aggregated topic list was generated. Email 2 asked each panelist to review the scope of the list and state his or her recommendation for each topic in the aggregated list. Later iterations of email interviews were organized to evolve the list until all experts agreed on the list. Because of the logistic complexity of coordinating the large panel, we took a grow-shrink approach. In the growing phase, all suggested items were included, even an item suggested by only 1 panelist. In the shrinking phase, any item opposed by a panelist was excluded. As it turned out, most items were initially suggested by a panelist but seconded by other panelists, suggesting the importance of the group effort for covering most important topics.

The practical steps were developed by machine learning experts in their respective areas and finally approved by the panel. During the process, the panelists consulted extensively the broad literature on machine learning and predictive model in particular [16-18].

## Results

A total of 4 iterations of emails resulted in the final form of the guidelines. Email 1 generated diverse responses in terms of topics. However the final scope was generally agreed upon. For email 2, most panelists commented on only a subset of topics (mostly the ones suggested by themselves). No recommendations generated significant disagreement except for minor wording decisions and quantifying conditions.

**Figure 1:**
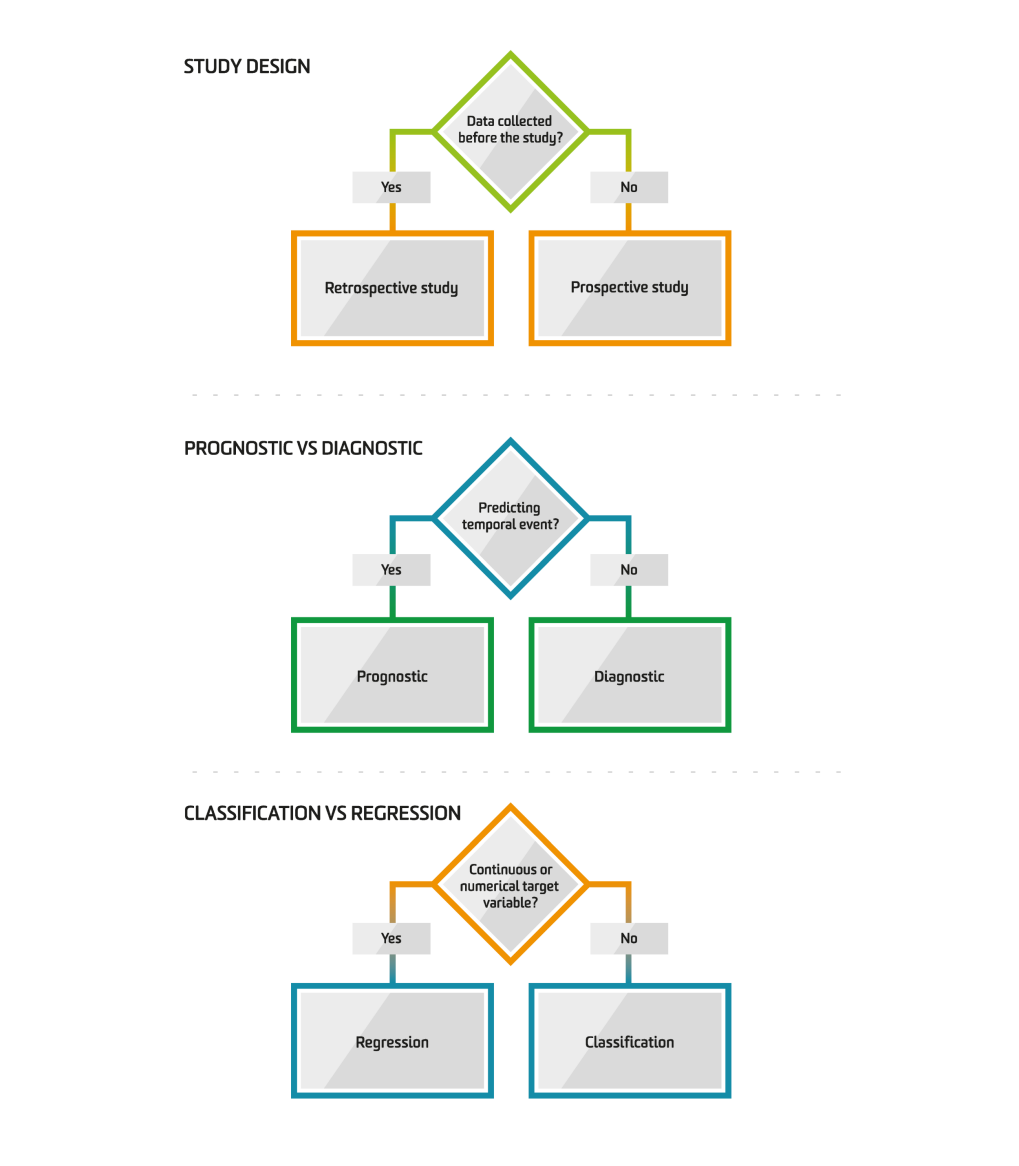
Steps to identify the prediction problem.

**Figure 2:**
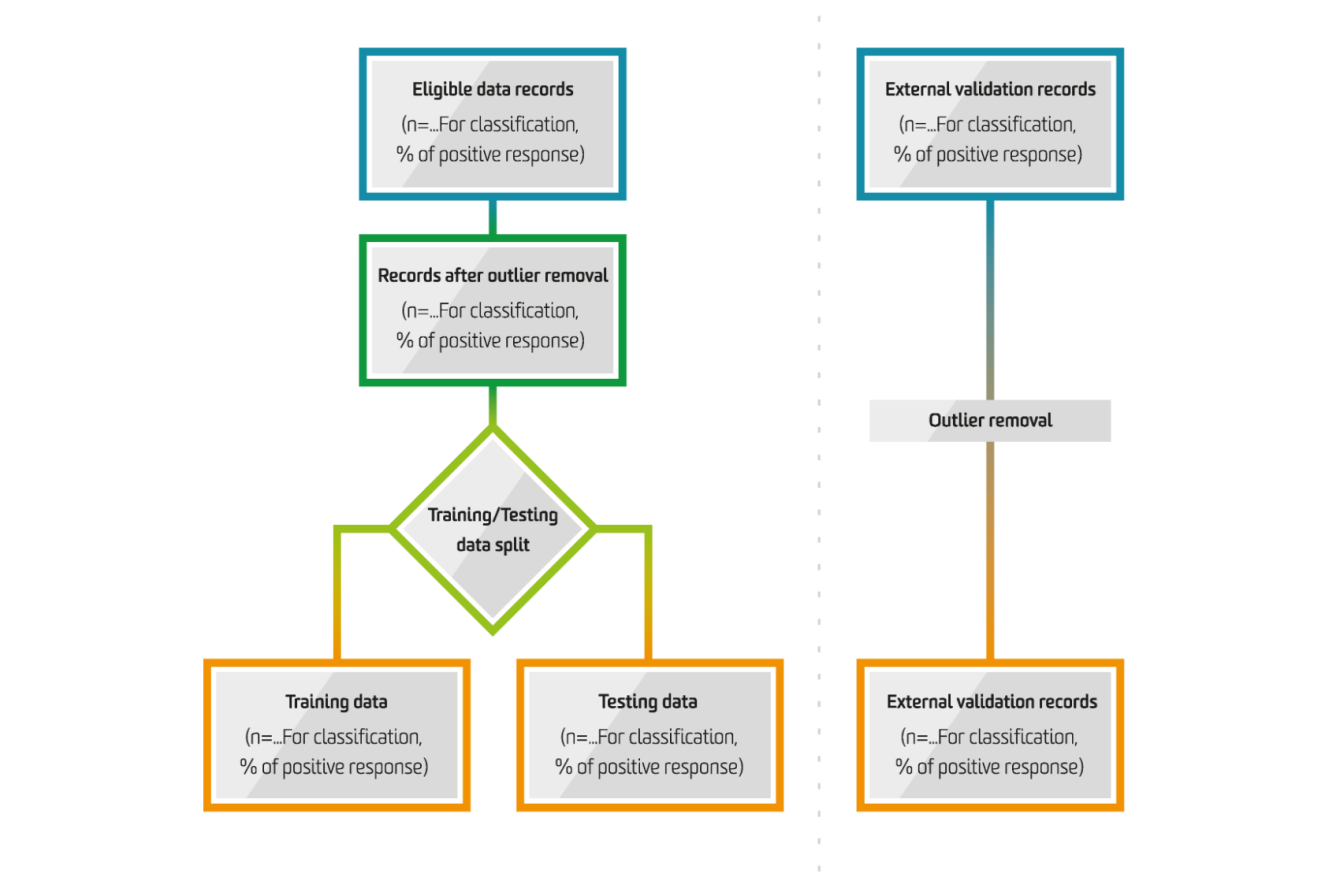
Information flow in the predictive modelling process.

The final results included a list of reporting items (Tables 1-5,Textboxes 1-4, and Figure 1) and a template flowchart for reporting data used for training and testing predictive models, including both internal validation and external validation (Figure 2).

Recognizing the broad meaning of the term “machine learning,” we distinguish essential items from desirable items (using appropriate footnotes in the tables). The essential items should be included in any report, unless there is a strong reason indicating otherwise; the desirable items should be reported whenever applicable.

**Table 1:** Items to include when reporting predictive models in biomedical research: title and abstract.

Item number	Section	Topic	Checklist item
1	Title	Nature of study	Identify the report as introducing a predictive model
2	Abstract	Structured summary	Background Objectives Data sources Performance metrics of the predictive model or models, in both point estimates and confidence intervals Conclusion including the practical value of the developed predictive model or models

**Table 2:** Items to include when reporting predictive models in biomedical research: introduction section.

Item number	Topic	Checklist item
3	Rationale	Identify the clinical goal Review the current practice and prediction accuracy of any existing models
4	Objectives	State the nature of study being predictive modeling, defining the target of prediction Identify how the prediction problem may benefit the clinical goal

**Table 3:** Items to include when reporting predictive models in biomedical research: methods section.

Item number	Topic	Checklist item
5	Describe the setting	Identify the clinical setting for the target predictive model. Identify the modeling context in terms of facility type, size, volume, and duration of available data.
6	Define the prediction problem	Define a measurement for the prediction goal (per patient or per hospitalization or per type of outcome). Determine that the study is retrospective or prospective.^a^ Identify the problem to be prognostic or diagnostic. Determine the form of the prediction model: (1) classification if the target variable is categorical, (2) regression if the target variable is continuous, (3) survival prediction if the target variable is the time to an event. Translate survival prediction into a regression problem, with the target measured over a temporal window following the time of prediction. Explain practical costs of prediction errors (eg, implications of underdiagnosis or overdiagnosis). Defining quality metrics for prediction models.^b^ Define the success criteria for prediction (eg, based on metrics in internal validation or external validation in the context of the clinical problem).
7	Prepare data for model building	Identify relevant data sources and quote the ethics approval number for data access. State the inclusion and exclusion criteria for data. Describe the time span of data and the sample or cohort size. Define the observational units on which the response variable and predictor variables are defined. Define the predictor variables. Extra caution is needed to prevent information leakage from the response variable to predictor variables.^c^ Describe the data preprocessing performed, including data cleaning and transformation. Remove outliers with impossible or extreme responses; state any criteria used for outlier removal. State how missing values were handled. Describe the basic statistics of the dataset, particularly of the response variable. These include the ratio of positive to negative classes for a classification problem and the distribution of the response variable for regression problem. Define the model validation strategies. Internal validation is the minimum requirement; external validation should also be performed whenever possible. Specify the internal validation strategy. Common methods include random split, time-based split, and patient-based split. Define the validation metrics. For regression problems, the normalized root-mean-square error should be used. For classification problems, the metrics should include sensitivity, specificity, positive predictive value, negative predictive value, area under the ROC^d^ curve, and calibration plot [19].^e^ For retrospective studies, split the data into a derivation set and a validation set. For prospective studies, define the starting time for validation data collection.
8	Build the predictive model	Identify independent variables that predominantly take a single value (eg, being zero 99% of the time). Identify and remove redundant independent variables. Identify the independent variables that may suffer from the perfect separation problem.^f^ Report the number of independent variables, the number of positive examples, and the number of negative examples. Assess whether sufficient data are available for a good fit of the model. In particular, for classification, there should be a sufficient number of observations in both positive and negative classes. Determine a set of candidate modeling techniques (eg, logistic regression, random forest, or deep learning). If only one type of model was used, justify the decision for using that model.^g^ Define the performance metrics to select the best model. Specify the model selection strategy. Common methods include K-fold validation or bootstrap to estimate the lost function on a grid of candidate parameter values. For K-fold validation, proper stratification by the response variable is needed.^h^ For model selection, include discussion on (1) balance between model accuracy and model simplicity or interpretability, and (2) the familiarity with the modeling techniques of the end user.^i^

^a^See Figure 1.

^b^See some examples in Multimedia Appendix 2.

^c^See Textbox 1.

^d^ROC: receiver operating characteristic.

^e^Also see Textbox 2.

^f^See Textbox 3.

^g^See Multimedia Appendix 1 for some common methods and their strengths and limitations.

^h^See Textbox 4.

^i^A desirable but not mandatory item.

**Table 4:** Items to include when reporting predictive models in biomedical research: results section.

Item number	Topic	Checklist item
9	Report the final model and performance	Report the predictive performance of the final model in terms of the validation metrics specified in the methods section. If possible, report the parameter estimates in the model and their confidence intervals. When the direct calculation of confidence intervals is not possible, report nonparametric estimates from bootstrap samples. Comparison with other models in the literature should be based on confidence intervals. Interpretation of the final model. If possible, report what variables were shown to be predictive of the response variable. State which subpopulation has the best prediction and which subpopulation is most difficult to predict.

**Table 5:** Items to include when reporting predictive models in biomedical research: discussion section.

Item number	Topic	Checklist item
10	Clinical implications	Report the clinical implications derived from the obtained predictive performance. For example, report the dollar amount that could be saved with better prediction. How many patients could benefit from a care model leveraging the model prediction? And to what extent?
11	Limitations of the model	Discuss the following potential limitations: • Assumed input and output data format • Potential pitfalls in interpreting the model^a^ • Potential bias of the data used in modeling • Generalizability of the data
12	Unexpected results during the experiments	Report unexpected signs of coefficients, indicating collinearity or complex interaction between predictor variables^a^

^a^Desirable but not mandatory items.

**Data leakage problem.**

Leakage refers to the unintended use of known information as unknown. There are two kinds of leakage: outcome leakage and validation leakage. In outcome leakage, independent variables incorporate elements that can be used to easily infer outcomes. For example, a risk factor that spans into the future may be used to predict the future itself. In the validation leakage, ground truth from the training set may propagate to the validation set. For example, when the same patient is used in both training and validation, the future outcome in the training may overlap with the future outcome in the validation. In both leakage cases, the performance obtained is overoptimistic.

**Calibration.**

Calibration of a prediction model refers to the agreement between the predictions made by the model and the observed outcomes. As an example, if the prediction model predicts 70% risk of mortality in the next 1 year for a patient with lung cancer, then the model is well calibrated if in our dataset approximately 70% of patients with lung cancer die within the next 1 year.

Often, the regularized prediction may create bias in a model. Therefore, it is advisable to check for the calibration. In the case of regression models, the calibration can be easily assessed graphically by marking prediction scores on the x-axis and the true outcomes on the y-axis. In the case of binary classification, the y-axis has only 0 and 1 values; however, smoothing techniques such as LOESS algorithm may be used to estimate the observed probabilities for the outcomes. In a more systematic way, one can perform the Hosmer-Lemeshow test to measure the goodness of fit of the model. The test assesses whether the observed event rates match the predicted event rates in subgroups of the model population.

**Perfect separation problem.**

When a categorical predictor variable can take an uncommon value, there may be only a small number of observations having that value. In a classification problem, these few observations by chance may have the same response value. Such “perfect” predictors may cause overfitting, especially when tree-based models are used. Therefore, special treatment is required.

One conservative approach is to remove all dummy variables corresponding to rare categories. We recommend a cutoff of 10 observations.

For modeling methods with feature selection built in, an alternative approach is to first fit a model with all independent variables. If the resulting model is only marginally influenced by the rare categories, then the model can be kept. Otherwise, the rare categories showing high “importance” score are removed and the model refitted.

**K-fold cross-validation.**

K-fold validation refers to the practice of splitting the derivation data into K equal parts. The model is then trained on K−1 parts and validated on the remaining part. The process is repeated K times. The average results for K-folds are then reported. For small classes and rare categorical factors, stratified K-fold splitting should be used to ensure the equal presence of these classes and factors in each fold.

## Discussion

We have generated a set of guidelines that will enable correct application of machine learning models and consistent reporting of model specifications and results in biomedical research.

Because of the broad range of machine learning methods that can be used in biomedical applications, we involved a large number of stakeholders, as either developers of machine learning methods or users of these methods in biomedicine research.

The guidelines here cover most popular machine learning methods appearing in biomedical studies. We believe that such guidelines will accelerate the adoption of big data analysis, particularly with machine learning methods, in the biomedical research community.

Although the proposed guidelines result from a voluntary effort without dedicated funding support, we still managed to assemble a panel of researchers from multiple disciplines, multiple institutions, and multiple continents. We hope the guidelines can result in more people contributing their knowledge and experience in the discussion.

As machine learning is a rapidly developing research area, the guidelines are not expected to cover every aspect of the modeling process. The guidelines are expected to evolve as research in biomedicine and machine learning progresses.

## Multimedia Appendix 1

Guidelines for fitting some common predictive models.

## Multimedia Appendix 2

Glossary.
