# Supplementary material for: Guidelines for Developing and Reporting Machine Learning Predictive Models in Biomedical Research: A Multidisciplinary View
Source: J Med Internet Res. 2016 Dec 16;18(12):e323. doi: 10.2196/jmir.5870 (PMC5238707; doi:10.2196/jmir.5870)
Supplement: Supplementary file 1 [file jmir_v18i12e323_app1.pdf]

Decision tree

A decision tree is a rule-based model strongly influenced by earlier work of Quinlan [1, 2]. Reviews of decision trees can be found at [3-5]. Decision tree, in general, does not have the best predictive accuracy compared with some other alternatives described below. However, it has the advantage in interpretability, with a format consistent with many clinical pathways.

Biomedical applications of decision trees are abundant (for example [6-9]). Common pitfalls of applying decision trees include overgrowing a tree with too few observations at leaf nodes. Such mistakes can be easily avoided by using a standard software package with sensible default values. We recommend that the minimum terminal node size should be at least 20.

Software packages	R: tree, rpart<br>Python scikits-learn: tree.DecisionTreeClassifier
Primary tuning parameter	complexity parameter cp: a percentage defining the minimum fit improvement at each node split

Random Forest

Random forest [10] is a popular predictive model that compared with other methods, generally produces very accurate prediction even without any tuning. Reviews of random forest can be found at [11, 12]. Random forest often can produce very accurate predictions with little feature engineering. It can also produce an “importance” ranking among all predictors. However, the models are in general not easily interpretable.

Biomedical applications of random forests have become more popular in recent years, especially in areas with high-dimensional data (e.g., genetic association studies) [13-15]. Common pitfalls of applying random forest include not optimizing the number of trees and insufficient randomization during the construction of base trees. Such mistakes are less likely to occur when using a standard statistical package and selecting the best tuning parameters. We recommend the number of trees in the randomForest should be at least 25 and ideally 500 or more. The minimum number of observations in each splitting node and leaf node should be 20 or more.

Software packages	R: randomForest

	Python scikits-learn: RandomForestClassifier
Primary tuning parameter	mtry: the number of randomly selected variables for comparison at each node split (<= number of independent variables)

Lasso Regression

Lasso is a regression regularization method introduced by Tibshirani [16]. Reviews of lasso regression can be found at [17-19]. Lasso regression is often used to fit a linear model when independent variables may be highly correlated. Compared to a traditional penalisation method such as ridge regularisation, lasso has the advantage of returning a sparse model (with fewer nonzero coefficients), and hence better model interpretability. Lasso in general provides a prediction bias towards zero, which may not be appropriate in some applications.

Lasso and its variants are popular in biomedical applications where a regression model is desired [20-22].

Common pitfalls of applying lasso regression include not tuning the shrinkage fraction. This can be done through cross-validation; most statistical packages provide tools for cross-validation.

Software packages	R: elasticnet, lars, glmnet<br>Python scikits-learn: linear_model.Lasso
Primary tuning parameter	fraction: the degree of coefficient shrinkage (0-1)

Gradient Boosting Machines

Gradient boosting [23] is based on the ensemble idea similar to Random forest. Reviews of gradient boosting can be found at [24-26]. Gradient boosting is generally considered to have performance comparable to Random forest. Compared to random forest, it has more tuning parameters. However, with most statistical packages for gradient boosting, default parameters (with small learning rate) will generate very stable results.

Like random forest, gradient boosting is also used many recent applications with high-dimensional data [27, 28]. One common pitfall of applying gradient boosting is to use a large learning rate without a proper stopping criterion, hence causing overfitting. We recommend a learning rate no greater than 0.1.

Software packages	R: gbm<br>Python scikits-learn:

	ensemble.GradientBoostingClassifier
Primary tuning parameter	n.trees: number of trees (boosting iterations)<br>interaction.depth: maximum depth for variable interaction (normally 1 to 6)<br>shrinkage: also known as learning rate (normally set to a small number such as 0.1)<br>n.minobsinnode: minimum number of observations in a terminal node (normally fixed to a number like 20)

Support vector machines

Support Vector Machines [29, 30] are a family of machine learning techniques based on the concept of structural risk minimization that was originally introduced by Vapnik [30]. They can be used for classification [31], regression [32] and density estimation [33], among other applications [34-37].

Support vector machines can produce very accurate predictions, have relatively few parameters that require tuning, and are largely insensitive to the dimensionality of the data. However the models produced are generally not readily interpretable, and model selection is biased toward "simple" models, which may not be appropriate in some applications. Common pitfalls when using SVMs include not optimising the tuning parameters appropriately and failing to test appropriate kernel functions. We recommend that at minimum RBF and polynomial kernels (to order 3) should be tested. Cross-validation may be used to select tuning parameters.

Software packages	R: e1071<br>Python scikits-learn: svm.SVC, svm.SVR<br>Stand-alone Software packages: SVMlight, LibSVM, SVMHeavy
Primary tuning parameter	C: controls the tradeoff between empirical risk minimization and regularisation. Large C values will favour empirical risk minimization, which may cause over-fitting, while small C values will

	favour regularisation and model simplicity, which may lead to under-fitting.<br>Kernel parameters: depending on the kernel selected there may be arbitrarily many parameters (or none at all) to select. For standard<br>kernels:<br>RBF kernel: single continuous parameter $\gamma > 0$ controls the width of the RBF.<br>Polynomial kernel: single discrete parameter d = 1, 2, 3, ... selects the degree of the polynomial.

References

1. Quinlan, J.R., *Simplifying decision trees*. International journal of man-machine studies, 1987. **27**(3): p. 221-234.
2. Quinlan, J.R., *Induction of decision trees*. Machine learning, 1986. **1**(1): p. 81-106.
3. Podgorelec, V., et al., *Decision trees: an overview and their use in medicine*. Journal of medical systems, 2002. **26**(5): p. 445-463.
4. Kotsiantis, S.B., *Decision trees: a recent overview*. Artificial Intelligence Review, 2013. **39**(4): p. 261-283.
5. Kingsford, C. and S.L. Salzberg, *What are decision trees?* Nat Biotechnol, 2008. **26**(9): p. 1011-3.
6. Luo, W. and M. Gallagher. *Unsupervised DRG upcoding detection in healthcare databases*. in *Data Mining Workshops (ICDMW), 2010 IEEE International Conference on*. 2010. IEEE.
7. Siddique, J., et al., *Applying Classification Trees to Hospital Administrative Data to Identify Patients with Lower Gastrointestinal Bleeding*. PLoS One, 2015. **10**(9): p. e0138987.
8. Bae, S.M., S.A. Lee, and S.H. Lee, *Prediction by data mining, of suicide attempts in Korean adolescents: a national study*. Neuropsychiatr Dis Treat, 2015. **11**: p. 2367-75.
9. Satomi, J., et al., *Predictability of the future development of aggressive behavior of cranial dural arteriovenous fistulas based on decision tree analysis*. J Neurosurg, 2015. **123**(1): p. 86-90.
10. Breiman, L., *Random Forests*. Machine Learning, 2001. **45**(1): p. 5-32.
11. Boulesteix, A.L., et al., *Overview of random forest methodology and practical guidance with emphasis on computational biology and bioinformatics*. Wiley Interdisciplinary Reviews: Data Mining and Knowledge Discovery, 2012. **2**(6): p. 493-507.
12. Strobl, C., J. Malley, and G. Tutz, *An introduction to recursive partitioning: rationale, application, and characteristics of classification and regression trees, bagging, and random forests*. Psychol Methods, 2009. **14**(4): p. 323-48.
13. Touw, W.G., et al., *Data mining in the Life Sciences with Random Forest: a walk in the park or lost in the jungle?* Brief Bioinform, 2013. **14**(3): p. 315-26.

14. Asaoka, R., et al., *Combining multiple HRT parameters using the 'Random Forests' method improves the diagnostic accuracy of glaucoma in emmetropic and highly myopic eyes.* Invest Ophthalmol Vis Sci, 2014. **55**(4): p. 2482-90.
15. Yoshida, T., et al., *Discriminating between glaucoma and normal eyes using optical coherence tomography and the 'Random Forests' classifier.* PLoS One, 2014. **9**(8): p. e106117.
16. Tibshirani, R., *Regression shrinkage and selection via the lasso.* Journal of the Royal Statistical Society. Series B (Methodological), 1996: p. 267-288.
17. Tibshirani, R., *Regression shrinkage and selection via the lasso: a retrospective.* Journal of the Royal Statistical Society: Series B (Statistical Methodology), 2011. **73**(3): p. 273-282.
18. Vidaurre, D., C. Bielza, and P. Larrañaga, *A survey of L1 regression.* International Statistical Review, 2013. **81**(3): p. 361-387.
19. Hesterberg, T., et al., *Least angle and ℓ1 penalized regression: A review.* Statistics Surveys, 2008. **2**: p. 61-93.
20. Fujino, Y., et al., *Applying "Lasso" Regression to Predict Future Visual Field Progression in Glaucoma Patients.* Invest Ophthalmol Vis Sci, 2015. **56**(4): p. 2334-9.
21. Shimizu, Y., et al., *Toward Probabilistic Diagnosis and Understanding of Depression Based on Functional MRI Data Analysis with Logistic Group LASSO.* PLoS One, 2015. **10**(5): p. e0123524.
22. Lee, T.F., et al., *Using multivariate regression model with least absolute shrinkage and selection operator (LASSO) to predict the incidence of Xerostomia after intensity-modulated radiotherapy for head and neck cancer.* PLoS One, 2014. **9**(2): p. e89700.
23. Friedman, J.H., *Stochastic gradient boosting*. Computational Statistics & Data Analysis, 2002. **38**(4): p. 367-378.
24. Natekin, A. and A. Knoll, *Gradient boosting machines, a tutorial.* Front Neurorobot, 2013. **7**: p. 21.
25. De'Ath, G., *Boosted trees for ecological modeling and prediction.* Ecology, 2007. **88**(1): p. 243-251.
26. Mayr, A., et al., *The evolution of boosting algorithms. From machine learning to statistical modelling*. Methods Inf Med, 2014. **53**(6): p. 419-27.
27. Ayaru, L., et al., *Prediction of Outcome in Acute Lower Gastrointestinal Bleeding Using Gradient Boosting*. PLoS One, 2015. **10**(7): p. e0132485.
28. Gonzalez-Recio, O., J.A. Jimenez-Montero, and R. Alenda, *The gradient boosting algorithm and random boosting for genome-assisted evaluation in large data sets.* J Dairy Sci, 2013. **96**(1): p. 614-24.
29. Cristianini, N. and J. Shawe-Taylor, *An Introduction to Support Vector Machines and other Kernel-Based Learning Methods*. 2005: Cambridge University Press.
30. Cortes, C. and V. Vapnik, *Support Vector Networks.* Machine Learning, 1995. **20**(3): p. 273-297.
31. Burges, C., *A Tutorial on Support Vector Machines for Pattern Recognition.* Knowledge Discovery and Data Mining, 1998. **2**(2): p. 121-167.
32. Smola, A. and B. Schölkopf, *A Tutorial on Support Vector Regression*. 1998.
33. Vapnik, V. and S. Mukherjee, *Support Vector Method for Multivariate Density Estimation*, in *Advances in Neural Information Processing Systems*. 2000, MIT Press.
34. Manevitz, L.M. and M. Yousef, *One-Class SVMs for Document Classification.* Journal of Machine Learning Research, 2001. **2**: p. 139-154.
35. Tsochantaridis, I., T.a.T. Hofmann, and Y. Altun. *Support vector machine learning for interdependent<p>and structured output spaces*. in *Proceedings of the twenty-first international<p>conference on Machine learning*.

36. Shilton, A., D. Lai, and P. Marimuthu, *A Division Algebraic Framework for Multi-Dimensional Support Vector Regression.* IEEE Transactions on Systems, Man and Cybernetics, Part B, 2010. **40**(2): p. 517-528.
37. Shashua, A. and A. Levin, *Taxonomy of Large Margin Principle Algorithms for<p>Ordinal Regression Problems*. 2002.
