# Supplementary material for: Guidelines for Developing and Reporting Machine Learning Predictive Models in Biomedical Research: A Multidisciplinary View
Source: J Med Internet Res. 2016 Dec 16;18(12):e323. doi: 10.2196/jmir.5870 (PMC5238707; doi:10.2196/jmir.5870)
Supplement: Supplementary file 2 [file jmir_v18i12e323_app2.pdf]

Terms	Description
**Internal validation**	Validation of model using data from the same source as training
**External validation**	Validation of model using data from a source different from training
**Response variable**	Variable to be predicted. Also known as dependent variable
**Independent variable**	Variables used to predict the response variable. Also known as feature
**True positive (TP)**	Correct positive prediction
**True negative (TN)**	Correct negative prediction
**False positive (FP)**	Predicting positive, but actually negative
**False negative (FN)**	Predicting negative, but actually positive
**Sensitivity**	TP/(TP+FN)
**Specificity**	TN/(TN+FP)
**Positive predictive value (PPV)**	TP/(TP+FP)
**Negative predictive value (NPV)**	TN/(TN+FN)
**Receiver operating characteristic (ROC)**	A curve showing true positive rate (TPR) against the false positive rate (FPR) at various discrimination threshold settings
**Area under the ROC (AUC)**	The area under the ROC curve. It is often used to compare predictive models.
